# DAMBE6: New Tools for Microbial Genomics, Phylogenetics, and Molecular Evolution

**DOI:** 10.1093/jhered/esx033

**Published:** 2017-04-04

**Authors:** Xuhua Xia

**Affiliations:** 1 From the Department of Biology and Center for Advanced Research in Environmental Genomics, University of Ottawa, 30 Marie Curie, PO Box 450, Station A, Ottawa, ON K1N 6N5, Canada.

**Keywords:** Bioinformatics and computational genetics, Molecular systematics and phylogenetics, bioinformatics, genomics, index of translation elongation, phylogenetic analysis based on pairwise alignment, translation initiation analysis

## Abstract

DAMBE is a comprehensive software workbench for data analysis in molecular biology, phylogenetics, and evolution. Several important new functions have been added since version 5 of DAMBE: 1) comprehensive genomic profiling of translation initiation efficiency of different genes in different prokaryotic species, 2) a new index of translation elongation (I_TE_) that takes into account both tRNA-mediated selection and background mutation on codon–anticodon adaptation, 3) a new and accurate phylogenetic approach based on pairwise alignment only, which is useful for highly divergent sequences from which a reliable multiple sequence alignment is difficult to obtain. Many other functions have been updated and improved including PWM for motif characterization, Gibbs sampler for de novo motif discovery, hidden Markov models for protein secondary structure prediction, self-organizing map for nonlinear clustering of transcriptomic data, comprehensive sequence alignment, and phylogenetic functions. DAMBE features a graphic, user-friendly and intuitive interface, and is freely available from http://dambe.bio.uottawa.ca.

DAMBE (Data analysis for molecular biology and evolution) is a comprehensive software package for sequence manipulation and analysis featuring a user-friendly interface and a variety of analytical functions in bioinformatics, phylogenetics, and descriptive and comparative genomics. It is often listed as one of the most widely used software packages in molecular phylogenetics (Salemi and Vandamme 2003; Felsenstein 2004; Lemey, et al. 2009). Version 6 of DAMBE (DAMBE6) added several new functions in genomic evolution and phylogenetics since DAMBE5 (Xia 2013) and updated and improved a number of existing functions.

## Genomic Profiling of Translation Initiation Signal in Prokaryotic mRNA

Translation initiation is often rate-limiting in bacteria and in bacteriophage (Liljenstrom and von Heijne 1987; Bulmer 1991; Xia 2007c; Xia et al. 2007; Kudla et al. 2009; Tuller et al. 2010; Prabhakaran et al. 2015). Translation initiation signals on mRNA in prokaryotes include the start codon decoded by fMet-tRNA^fMet^ and Shine–Dalgarno sequence (SD) binding to the anti-SD (aSD) sequences at the 3′ end of small subunit ribosomal RNA (ssu rRNA) (Shine and Dalgarno 1974; Hui and de Boer 1987).

### What Is the Optimal SD/aSD Pairing?

**Figure 1.**
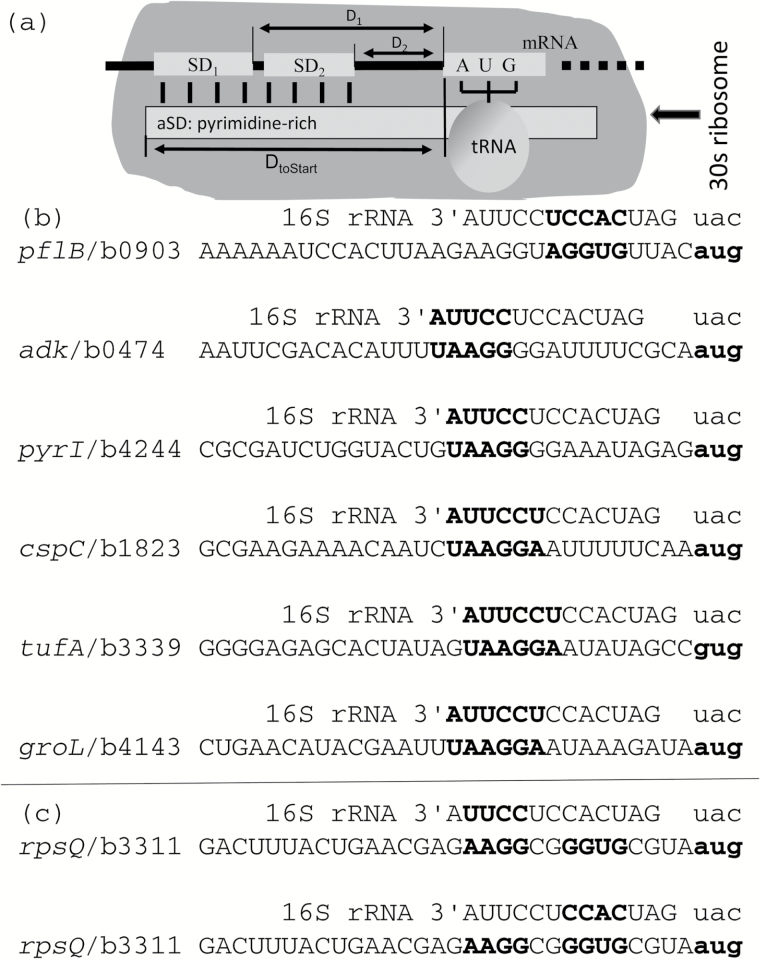
Key features of interacting components for juxtaposing the start codon against the anticodon of the initiating fMet-tRNA^fMet^. (**a**) A model of SD/aSD pairing. Two different SDs on mRNA (SD_1_ and SD_2_), with different distances (D_1_ and D_2_, respectively) to the initiation codon AUG can both properly align AUG against the tRNA. The 2 different SD/aSD pairings result in the same D_toStart_, defined as the distance between th’e 3′ end of ssu rRNA to the start codon. (**b**) A sample of SD/aSD pairing from highly expressed *Escherichia coli* genes, with the start codon and the tRNA anticodon in small case. (**c**) One example of a highly expressed gene (*rpsQ*) with 2 putative SDs (**c**). Gene IDs are in the form of “gene name/Locus_tag”. SDs in (b) and (c) differ in sequence and distance to the start codon, but they all have similar D_toStart_. A change in D_toStart_ will lead to misalignment of start codon and tRNA anticodon.

I will first clarify what constitute a good SD/aSD pairing. Structural determination (Milon et al. 2012) showed that fMet-tRNA^fMet^ and translation initiation factors can bind to 30S ribosome synergistically (binding of one facilitates the binding of others). The function of SD/aSD binding is to juxtapose the start codon against anticodon of fMet-tRNA^fMet^ (Figure 1a). While many genes have their SD being AGGAGGU or part of it, many have different SDs (Figure 1b). Each SD has its specific optimal distance (D) between the SD and start codon, for example, the optimal D is D_1_ for SD_1_ and D_2_ for SD_2_ in Figure 1a. One real case involves *Escherichia coli rpsQ* gene (Figure 1c) which has 2 putative SDs, AAGG and GGUG (Figure 1c). However, we note that the 2 SDs in Figure 1a have the same D_toStart_ defined as the distance between the 3′ end of ssu rRNA and the start codon (Figure 1a). D_toStart_ is strongly constrained within a narrow range in a variety of bacterial species from the gram-negative *E. coli* to the gram-positive *Bacillus subtilis*, suggesting that D_toStart_ is a better and more general index for measuring optimal positioning of SD/aSD pairing than D_1_ or D_2_ in Figure 1a (because D_1_ or D_2_ are SD-specific). The 2 putative SDs in the *rpsQ* gene (Figure 1c) bind to different aSDs but both have similar D_toStart_ values (15 and 14, respectively, Figure 1c). It is meaningless to state that the optimal distance between an SD and start codon is 5 or 10 nucleotides (nt) without specifying what SD is. An SD can be very close to the start codon, as in the case of *pflB* with only 4 nt in between (Figure 1) or far apart as in the case of *adk* with 11 nt in between (Figure 1b). What is common among all of them is that they all have similar D_toStart_ (Figure 1b, c).

**Figure 2.**
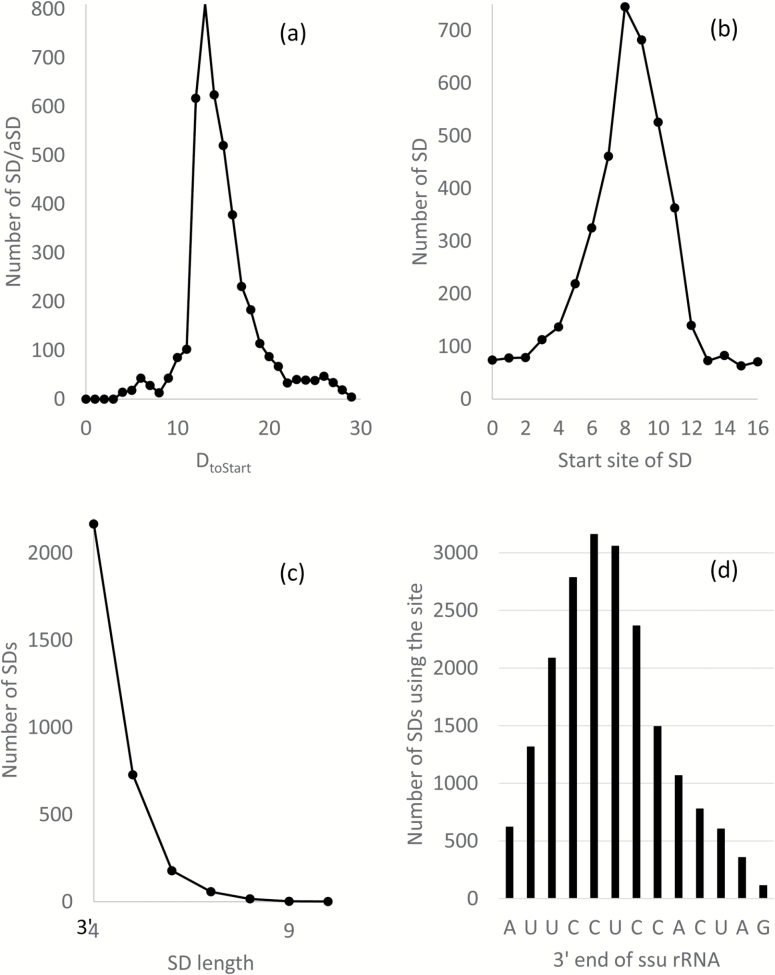
Summary statistics produced from DAMBE on SD/aSD pairing in *Escherichia coli* between 20 nt upstream of start codon and 13 nt at 3′ end of ssu rRNA, with minimum SD length equal to 4. (**a**) D_toStart_ is strongly constrained within a narrow range. (**b**) Most SDs are located within a narrow range upstream of the start codon. The start codon is at sites 21–23. (**c**) The 13 nt at 3′ end of *E. coli* ssu rRNA are differentially involved in SD/aSD pairing. A nucleotide substitution at the CU dinucleotide at sites 5 and 6 would affect more than 3000 SD/aSD pairings. (**d**) Most SDs are only 4 nt long, although longer SDs are often found to be more efficient in translation initiation.

Given an annotated prokaryotic genome, DAMBE can 1) extract the sequence upstream of each coding sequences (CDSs), for example, 20 nt immediately upstream of the initiation codon, 2) identify the putative SDs in all protein-coding sequences, and 3) output a variety of summary statistics to show which gene has a strong and optimally positioned SD/aSD. This is illustrated with data from *E. coli* (Figure 2). Most *E. coli* SD/aSD matches have D_toStart_ = 13 (Figure 2a). The frequency increases sharply from D_toStart_ = 11 to D_toStart_ = 12, but decreases more gradually on the right side (Figure 2a). This feature is common among diverse bacterial species. Most *E. coli* SDs are confined within a narrow range within 20 nt upstream of the start codon (Figure 2b). Three most frequent *E. coli* SDs are AGGA, GGAG, and GAGG which overlap to form the longer and better known motif of AGGAGG (Table 1). An overwhelming majority of SDs are 4-nt long (Figure 2d), although many studies suggest that longer SDs are more efficient in localizing the start codon (Vimberg et al. 2007).

**Table 1.** Frequency of different SDs in *Escherichia coli* protein-coding genes, ordered according to their pairing position along the 3′ end of ssu rRNA

Putative SD	Count
GAUC	85
UGAU	191
UGAUC	11
GUGA	146
GUGAU	26
GUGAUC	10
GGUG	72
GGUGA	42
GGUGAU	11
GGUGAUC	2
AGGU	168
AGGUG	42
AGGUGA	27
AGGUGAU	7
AGGUGAUC	2
GAGG	377
GAGGU	152
GAGGUG	41
GAGGUGA	25
GAGGUGAU	4
GAGGUGAUC	1
GGAG	479
GGAGG	167
GGAGGU	35
GGAGGUG	10
GGAGGUGA	5
GGAGGUGAU	1
AGGA	409
AGGAG	288
AGGAGG	54
AGGAGGU	13
AGGAGGUG	5
AGGAGGUGAU	1
AAGG	239
AAGGA	256
AAGGAG	169
AAGGAGG	23
AAGGAGGU	5
AAGGAGGUGA	1
AAGGAGGUG	2
UAAG	222
UAAGG	109
UAAGGA	152
UAAGGAG	121
UAAGGAGG	10
UAAGGAGGUG	1

The 13 nt at the 3′ end of *E. coil* ssu RNA are differentially involved in SD/aSD, with some sites (e.g., UCCUC at sites 3–7) involved in SD/aSD pairing more frequently than others. A nucleotide substitution at one of these sites will affect SD/aSD pairing (and consequently translation initiation) of thousands of genes. We therefore expect these sites to be extremely conserved due to the constraints of so many genes. A corollary from this framework of reasoning is that other sites constrained by fewer SD/aSD pairing would be more tolerated.

Translation initiation of most prokaryotic genes requires well-positioned SD/aSD base-pairing, although SD/aSD base-pairing is not always essential for translation in *E. coli* (Melancon et al. 1990; Fargo et al. 1998) and *Chlamydomonas reinhardtii* chloroplasts (Fargo et al. 1998), and for translating leaderless genes that have no SD sequence (Sartorius-Neef and Pfeifer 2004). The strength and position of SD/aSD base-pairing do strongly affect translation initiation in many genes (Shine and Dalgarno 1974; Hui and de Boer 1987; de Smit and van Duin 1994; Olsthoorn et al. 1995; Vimberg et al. 2007; Osterman et al. 2013). The tools offered in DAMBE facilitate large-scale study of SD/aSD coevolution as different species do have different 3′ end of small subunit rRNA (3′ TAIL) demanding different SD/aSD pairing dynamics.

### Translation Initiation Signal and Secondary Structure

SD and the start codon constitutes key translation initiation signals on mRNA to be recognized by ribosomes and initiation tRNA, respectively. Having these signals embedded in secondary structure decreases translation initiation efficiency (de Smit and van Duin 1990, 1994; Nivinskas et al. 1999; Milon and Rodnina 2012; Milon et al. 2012; Osterman et al. 2013), especially in highly expressed genes.

**Figure 3.**
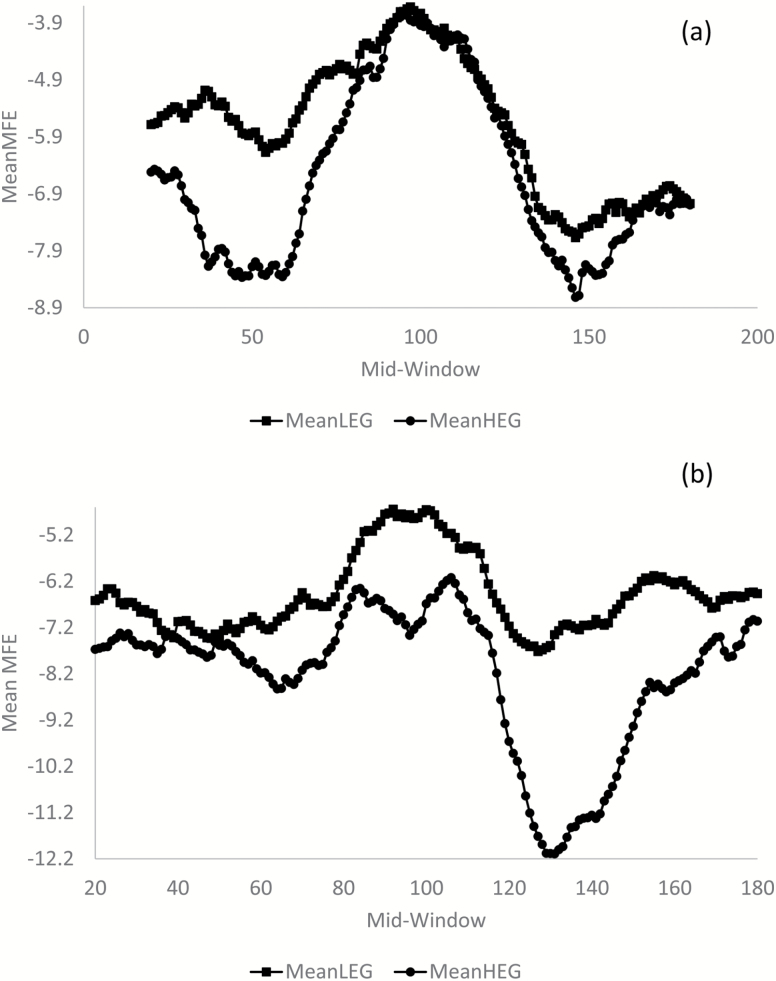
Change in MFE surrounding the start codon (**a**) and the stop codon (**b**). A sliding window of 40 nt is used. The start codon is at sites 101–103 in (a) and the stop codon is at sites 98–100 in (b). One thousand highly expressed genes (HEGs) and 1000 lowly expressed genes (LEGs) are used in contrasts.

DAMBE includes functions to extract CDSs and their upstream and downstream sequences, and can use a sliding window to compute minimum folding energy (MFE) which measures stability of local secondary structure (Hofacker 2003). The MFE profile shows a dramatic decrease in secondary structure around the start codon and the surrounding region, which is particularly pronounced in highly expressed genes (Figure 3a). A similar trend is observed in stop codons, but it is overshadowed by a much stronger increase in secondary structure stability about 30 nt downstream of the stop codon (Figure 3b), which is likely due to the hairpin involved in the rho-independent termination.

In prokaryotes, some genes are closely spaced with sequence configurations such as -AUGA- (where UGA is the stop codon of the upstream gene and AUG is the start codon of the downstream gene) and -UAAUG- (where UAA is the stop codon of the upstream gene and AUG is the start codon of the downstream gene). The patterns in Figure 3 are from genes with an intergenic sequence of at least 100 nt to avoid confounding the MFE pattern near the start codon and that at the stop codon. DAMBE can optionally include gene location information in the output sequence file.

## Index of Translation Elongation

Many gene-specific codon usage indices have already been formulated and improved, including CAI (Sharp and Li 1987; Xia 2007b), tAI (dos Reis et al. 2004) and several indices that are based on CDSs only, such as N_c_ (Wright 1990) and its improved versions (Novembre 2002; Sun et al. 2013). The first 2 have been used frequently as proxies for translation elongation efficiency, but they both have major problems (Xia 2007a, 2015).

The problem with tAI is that we often cannot infer which tRNA favors which synonymous codon. For example, inosine is expected to pair best with C and U, less with A (partly because of the bulky I/A pairing involving 2 purines), and not with G, but this is not true with tRNA^Val/IAC^ from rabbit liver which pairs better with GUG codon than with other synonymous codons (Jank et al. 1977; Mitra et al. 1977). Similarly, the *B. subtilis* genome codes a tRNA^Ala/GGC^ for decoding GCY codons, but the GCC codon which forms Watson–Crick base pair with the anticodon is not used as frequently as the GCU codon which wobble-pairs with the anticodon. Furthermore, codon–anticodon base pairing is known to be context-dependent (Lustig et al. 1989), for example, a wobble cmo^5^U in the anticodon of tRNA^Pro^, tRNA^Ala^, and tRNA^Val^ can read all 4 synonymous codons in the respective codon family, but the same cmo^5^U in tRNA^Thr^ cannot read C-ending codons (Nasvall et al. 2007). For this reason, the optimal codon usage is likely better approximated by the codon usage of highly expressed genes than what we can infer based on codon–anticodon pairing.

CAI also has problems (Xia 2007b, 2015). In particular, it ignores background mutation bias which can result in misinterpretation of tRNA-mediated selection. Take for example the Ala codon subfamily GCR (where R stands for either A or G). The frequencies of GCA and GCG in *E. coli* HEGs, as compiled and distributed with EMBOSS (Rice et al. 2000), are 1973 and 2654, respectively, which may lead one to think that *E. coli* translation machinery prefer GCG over GCA. However, GCA is relatively more frequent in *E. coli* HEGs than in *E. coli* non-HEGs. This suggests that mutation bias favors GCG, but tRNA-mediated selection favors GCA. This interpretation is corroborated by the *E. coli* genome encoding three tRNA^Arg^ genes for GCR codons, all with a UGC anticodon forming perfect Watson–Crick base pair with codon GCA.

**Figure 4.**
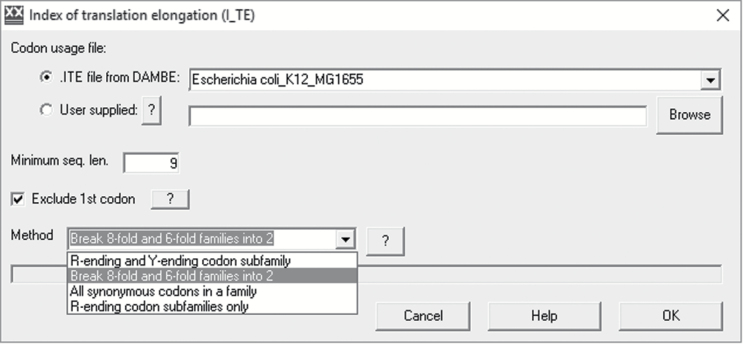
DAMBE’s interface for computing index of translation elongation (I_TE_) with 4 slightly different implementations. Codon usage tables for 120 species are included to facilitate computation, but users can supply their own codon usage tables.

DAMBE implements a new index of translation elongation (I_TE_) which incorporates both tRNA-mediated selection and background mutation bias and fits protein production better than CAI or tAI (Xia 2015). CAI is a special case of I_TE_ when background mutation bias is absent. There are 4 variations of I_TE_ with different treatment of synonymous codon families (Figure 4). The first is to treat R-ending and Y-ending codon groups as if they are separate codon families, with reasons for such a treatment outlined before (Xia 2015). The second (the default) is to separate compound 8-fold codon families into 2 separate 4-fold codon families, and 6-fold codon families into 2 codon families with 4 and 2 synonymous codons each. Such separation is reasonable because the 4-codon and 2-codon synonymous families are translated by different tRNAs. The third is to lump all synonymous codons into one codon family. The fourth is to use only R-ending codons because, in some species such as *E. coli*, codon bias is strong in R-ending synonymous codons but weak in Y-ending synonymous codons. I_TE_ has been used to facilitate studies on translation initiation and elongation in bacteriophages (Prabhakaran et al. 2015) and coevolution between stop codons and release factors in bacteria (Wei and Xia 2016; Wei et al. 2016).

## Molecular Phylogenetics Based on Pairwise Alignment Only

Pairwise sequence alignment (PSA) by dynamic programming is guaranteed to generate one of the optimal alignments, but multiple sequence alignment (MSA) by dynamic programming is often not practical. The commonly used progressive alignment along a guide tree often results in poor alignment for highly diverged sequences in spite of many iterations to update the guide tree and the alignment, plaguing all subsequent phylogenetic analysis. One way to avoid this problem is to use only PSA to reconstruct phylogenetic trees, which can only be done with distance-based methods. DAMBE implements such a phylogenetic method (PhyPA) based only on pairwise alignment (Xia 2016). I compared the accuracy of PhyPA against the combination of maximum likelihood method and MSA (the ML+MSA approach), using nucleotide, amino acid, and codon sequences simulated with different topologies and tree lengths. Surprisingly, the fast PhyPA method consistently outperforms the slow ML+MSA approach for highly diverged sequences even when all optimization options were turned on for the ML+MSA approach (Xia 2016). Only when sequences are not highly diverged (i.e., when a reliable MSA can be obtained) does the ML+MSA approach outperforms PhyPA. The PhyPA method implemented in DAMBE also includes 2 approaches making use of multi-gene data sets to derive phylogenetic support for subtrees equivalent to resampling techniques such as bootstrapping and jackknifing.

**Figure 5.**
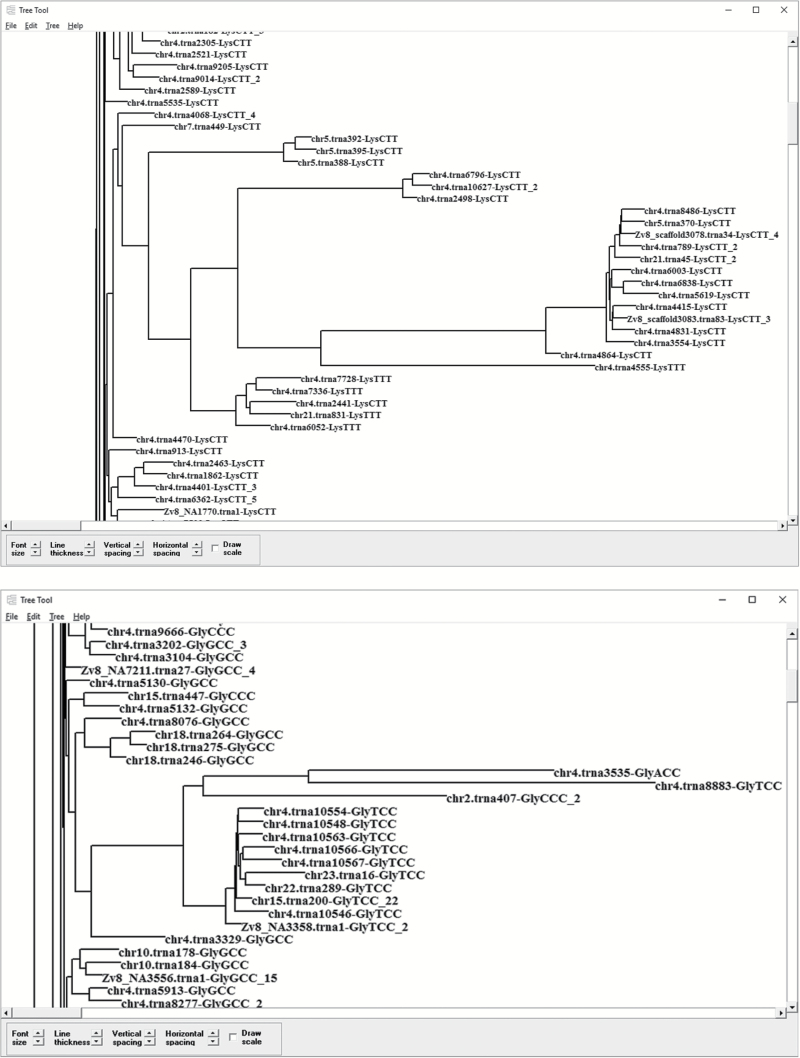
Use PhyPA to identify tRNA pseudogenes in zebrafish (*Danio rerio*) which has 12292 tRNA genes according to GtRNAdb (http://gtrnadb.ucsc.edu/). Top: partial tree from 1478 tRNA^Lys^ genes. Bottom partial tree from 1162 tRNA^Gly^ genes. tRNA genes with extraordinarily long branches are most likely pseudogenes. The tRNA sequence ID includes chromosome, amino acid, and anticodon.

PhyPA can also be used to characterize phylogenetic structure of gene families or identify pseudogenes. For example, after using PSI-BLAST to obtain hundreds or even thousands of sequences with remote homology, one can use PhyPA to reconstruct a phylogenetic tree based on pairwise alignment and the resulting tree structure will reflect the number of, and relationship among, gene families. PhyPA can also be used to quickly identify candidate pseudogenes. For example, zebrafish (*Danio rerio*) has 12292 tRNA genes according to GtRNAdb (http://gtrnadb.ucsc.edu/). Phylogenetic analysis with PhyPA revealed many tRNAs to have extraordinarily long branches and most likely are tRNA pseudogenes. This is exemplified by partial phylogenetic trees from 1478 tRNA^Lys^ genes (Figure 5, top) and from 1162 tRNA^Gly^ genes (Figure 5, bottom).

Many other functions in DAMBE have been updated and improved. A variety of statistical tests have been added to position weight matrix for motif characterization (Xia 2012) which has been applied in characterizing the splicing signal strength in yeast (Ma and Xia 2011) and vertebrates (Vlasschaert et al. 2015). The function for handling multiple files in a number of phylogenetic analyses is particularly useful with sequence simulation that includes indels. Such simulations often generate a large number of unaligned sequence files. With only a few clicks, DAMBE will be able to align all these files, reconstruct phylogenetic trees and compare the differences between the resulting trees and the true tree used in sequence simulation. Other functions that have been improved include hidden Markov models for protein secondary structure prediction, Gibbs sampler for de novo motif discovery, and self-organizing map for nonlinear clustering of transcriptomic data (Xia and Xie 2001; Xia 2007a, p. 231–250).

In short, DAMBE is a comprehensive software workbench in molecular biology, phylogenetics, and evolution, with new functions continuously added to empower researchers to perform leading-edge data analysis in prokaryotic genomic data to solve practical research problems. DAMBE is user-friendly with a variety of graphic functions, which makes it ideal not only for research, but also for teaching. DAMBE is available free of charge from http://dambe.bio.uottawa.ca, where a set of laboratory tutorials designed for teaching can be found. DAMBE is a Windows program, but may run on Linux and Macintosh computers.

## Funding

This work was supported by the Discovery Grant of Natural Science and Engineering Research Council of Canada (NSERC, RGPIN/261252–2013).
